# Impact of COVID-19 on tuberculosis detection and treatment in Baja California, México

**DOI:** 10.3389/fpubh.2022.921596

**Published:** 2022-07-22

**Authors:** Raquel Muñiz-Salazar, Tina Le, Jazmine Cuevas-Mota, Jesús Eduardo González-Fagoaga, Rogelio Zapata-Garibay, Paola Saritzia Ruiz-Tamayo, Javier Robles-Flores, Richard S. Garfein

**Affiliations:** ^1^Escuela de Ciencias de la Salud, Universidad Autónoma de Baja California, Ensenada, Mexico; ^2^Herbert Wertheim School of Public Health, University of California, San Diego, San Diego, CA, United States; ^3^Healthy Border Program, US-Mexico Border Heath Commission, Tijuana, Mexico; ^4^Facultad de Humanidades y Ciencias Sociales, Universidad Autónoma de Baja California, Tijuana, Mexico

**Keywords:** SARS-CoV-2, migration, *Mycobacterium tuberculosis*, qualitative analysis, incidence

## Abstract

In 2020, Mexico reported the lowest tuberculosis (TB) incidence on record, and it is unclear to what extent COVID-19 has impacted TB surveillance, diagnosis, and treatment. It is important to understand COVID-19's impact in Baja California (BC), which has the highest TB burden in Mexico. With the increasing number of migrants and asylum seekers arriving in BC, limited resources and crowded living conditions increase the risk of TB transmission. The purpose of this study was to assess the impact of COVID-19 on TB diagnosis and treatment in BC. We were also interested in health disparities experienced by migrants in BC. We conducted a mixed methods analysis using quantitative surveillance data obtained from the Mexico National TB Program (NTP) and qualitative data collected through in-depth interviews and focus group discussions with TB program directors and personnel in BC's four provincial health jurisdictions. Compared to the year prior, surveillance data from March 2020 - February 2021 revealed that TB incidence in BC declined by 30.9% and favorable TB outcomes (TB cure or treatment completion) declined by 49.8%. Elucidating differences by migrant status was complicated by the lack of standardized collection of migrant status by the NTP. Qualitative analysis revealed that TB diagnostic and treatment supplies and services became limited and disproportionately accessible across jurisdictions since the pandemic began; however, favorable adaptations were also reported, such as increased telemedicine use and streamlined care referral processes. Participants shared that migrant status is susceptible to misclassification and that TB care is difficult due to the transitory nature of migrants. This study did not identify major differences in TB service delivery or access between migrants and non-migrants in BC; however, migrant status was frequently missing. COVID-19 has overwhelmed health systems worldwide, disrupting timely TB diagnostic and treatment services, and potentially caused underdiagnosis of TB in BC. TB programs in BC should quickly restore essential services that were disrupted by COVID-19 while identifying and preserving beneficial program adaptations, such as telemedicine and streamlined care referral processes. Improved methods for documenting migrant status of TB cases are also needed.

## Introduction

COVID-19 was declared a global pandemic by the World Health Organization on March 11, 2020 (1). In total, 1,813,188 COVID-19 deaths were reported that year globally, although excess mortality statistics indicate this number should be closer to 3 million (1). Conversely, new TB case reports have declined 18% worldwide from 7.1 million in 2019 to 5.8 million in 2020; however, TB-related deaths increased from 1.4 million in 2019 to 1.5 million in 2020 (2). One reason for these discrepancies may be due to variations in health system capacity. For example, the percentage of registered deaths alone ranged from 98% in European countries to 10% in African counties, suggesting that healthcare services and case management have been disproportionately impacted by the pandemic (1, 3).

On March 23, 2020, Mexico implemented the National Campaign for Healthy Distance (Jornada Nacional de Sana Distancia), a public health intervention that suspended all non-essential in-person activities for 2 months (4). On June 1, 2020, the “new normality” phase started, which was a plan to resume economic, social, and educational activities in Mexico according to a risk assessment system with levels that resembled a traffic light (5). Health systems in Mexico prioritized COVID-19 mitigation efforts and were overwhelmed by high COVID-19 case rates, consequently interrupting other health services (6). By December 27, 2020, Mexico reported 1,372,243 official cases and 121,837 confirmed deaths—the fourth most COVID-19 deaths at the time, behind the United States, Brazil, and India (7). Conversely, in 2020, Mexico reported its lowest TB case count (16,752) and incidence rate (13.1 cases per 100,000 persons) on record (8). COVID-19 is believed to have disrupted TB surveillance and treatment nationally and regionally in BC, where TB is highly endemic.

BC has the highest burden of TB in Mexico, with approximately 2,000 active cases annually (9), increasing slowly during the last years. In addition, BC has historically received thousands of migrants annually, who are especially vulnerable to airborne diseases like COVID-19 and TB due to crowded living situations that impede physical distancing and a lack of resources to maintain good hygiene (10, 11). In 2018, due to the growing number of migrant caravans seeking asylum from Central America and the Caribbean regions to the United States (US), US immigration authorities implemented the “metering” system, requiring migrants to wait in Mexico up to several months while their petitions were processing (12, 13). By early April 2020, as a result of an unilateral US response to COVID-19 control management, there were 14,400 waitlisted asylum seekers in 11 Mexican border cities, with 67% of these asylum seekers waiting in Tijuana, BC (14). Despite the influx of migrants in the state, BC reported lower TB incidence in 2020 than 2019 (51.0 vs. 63.6 cases per 100,000 persons, respectively). It is crucial to evaluate how COVID-19 has affected TB in BC, especially among migrant populations due to their vulnerability and risk of transmission.

The purpose of this study was to assess changes in the NTP activities and performance during the COVID-19 pandemic and to describe the impact of these changes on the diagnosis and treatment of TB among migrant and non-migrant populations in BC, Mexico. Results from this study can be used to inform future TB policies and practices to overcome COVID-19 setbacks.

## Methods

### Study design

We conducted a mixed methods study in BC, Mexico in 2021. The study included a quantitative analysis of NTP data for TB cases diagnosed *before* (2019-2020) and *during* (2020-2021) the COVID-19 pandemic. Qualitative data were collected from individuals working at all levels of TB control in BC through focus group discussions and key informant interviews.

### Setting and participants

**Figure 1:**
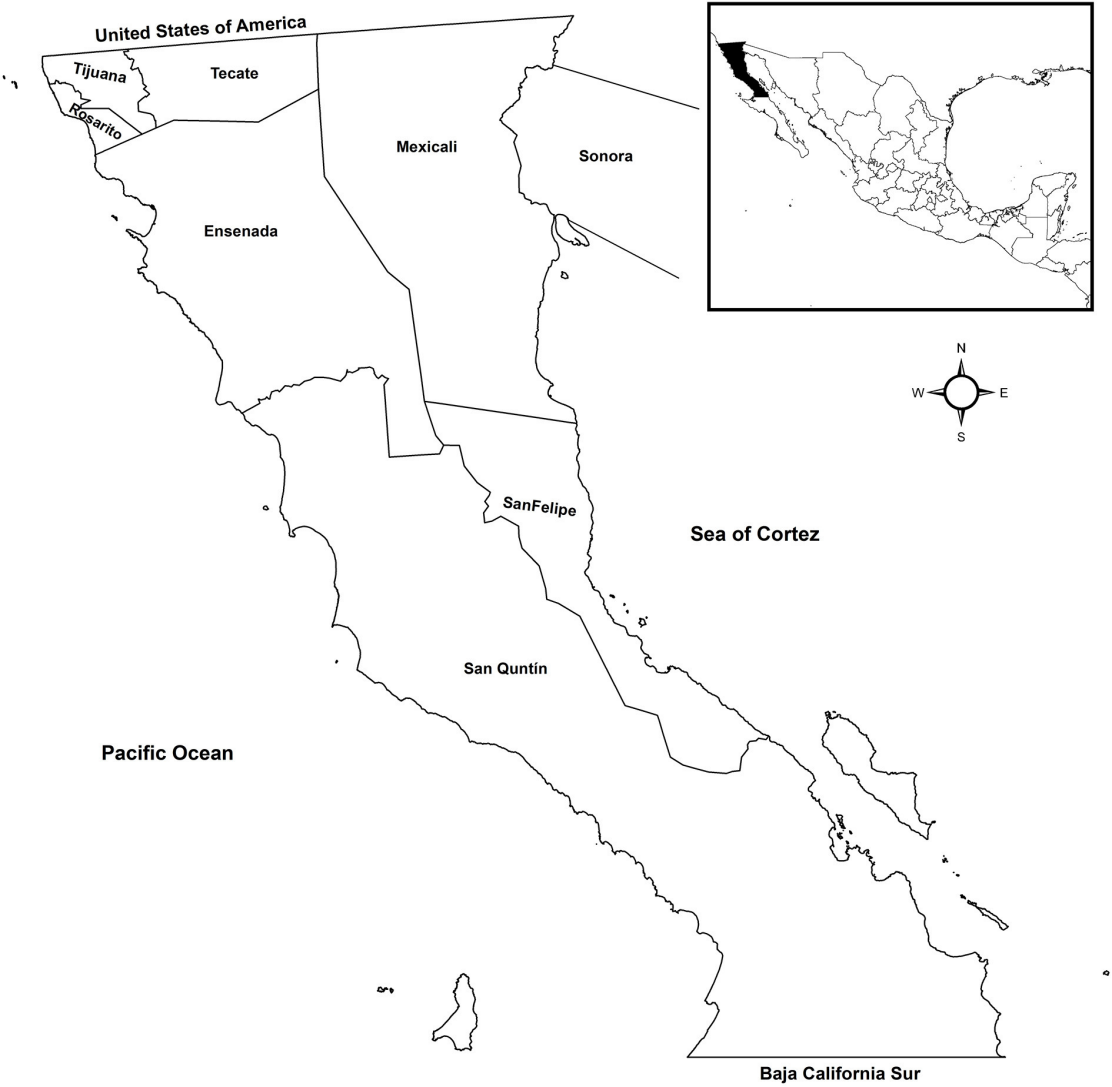
Map of Baja California, Mexico health jurisdictions included in the study. JI, Mexicali and San Felipe; JII, Tijuana, Rosarito, and Tecate; JIII, Ensenada; and JIV, San Quintin.

The state of BC is in northwestern Mexico, bounded to the north by California and Arizona in the US. BC is divided into seven municipalities grouped into four health jurisdictions designated as JI–JIV (Figure 1). TB diagnosis in BC is made using confirmatory sputum smear microscopy and/or positive cultures for *M. tuberculosis*. Confirmed TB cases are reported to the General Directorate of Epidemiology of Mexico.

TB program directors as well as physicians, nurses, laboratorians, and community health workers from the State Tuberculosis Program were included from each jurisdiction. Individuals were eligible to participate if they worked at least 20 h per week in the 6 months prior to recruitment. Participants were recruited by email using contact information provided by directors from each health facility. We obtained endorsement from the facility directors to increase participation among the facility personnel.

### Data collection

TB epidemiological data from 2019 to 2021 were extracted from Mexico's Ministry of Health management information system (https://tuberculosis.sinave.gob.mx/). These data were used to assess the impact of COVID-19 on TB treatment outcomes and case findings. The extracted data included TB case notifications and TB treatment outcomes (i.e., TB cure, treatment completion, treatment failure, incomplete treatment, relapse, loss to follow up, death, and conversion to drug resistance).

Focus groups were conducted to obtain information about strategies that were implemented during the COVID-19 lockdown to provide TB-related services to the community. Local health team officers, diagnostic supervisors, community health workers (promotoras) and nurses working in TB treatment centers participated in focus groups and excluded program directors to allow for open discussions. Focus groups consisted of 5–8 participants and lasted 60–90 min each. TB program directors participated in one-on-one in-depth interviews that lasted ~40 min. All focus groups and in-depth interviews were conducted between April 26, 2021 and May 31, 2021 using videoconferencing due to COVID-19 restrictions on in-person meetings.

Focus group topics included strategies implemented at each level of care for TB (i.e., case identification, diagnosis, notification, and treatment) in response to the COVID-19 pandemic, with emphasis on strategies targeting migrants. Participants were asked to share their perceptions on how well these strategies worked and what additional strategies could be implemented to improve services for migrants. Other topics included: diagnostic processes and challenges; loss to follow-up; TB treatment adherence; access to health care facilities; challenges providing TB care during the COVID-19 pandemic; problems faced by patients with TB and their caregivers; resources available at work to help manage the impact of the pandemic; and innovations or adaptations employed to mitigate the impact of COVID-19 on TB services. Focus group discussion questions were modified for interviews with TB program directors that covered the same topics.

We were unable to stratify the surveillance data by migration status, because the NTP does not collect that information.

### Data analysis

Quantitative analysis involved computing the monthly incidence of TB cases registered before (March 2019 to February 2020) and during (March 2020 to February 2021) the COVID-19 pandemic. *TB cure* and *treatment completion* were categorized as favorable TB outcomes, while *treatment failure, relapse, loss to follow-up, death, conversion to drug resistance*, and *incomplete treatment* were categorized as unfavorable treatment outcomes.

Focus group discussions and interviews were recorded and transcribed in Spanish (RMS, JEGF, PSRT, JFR and RZG). The transcripts were then translated into English by a single translator. Two bilingual co-investigators (RMS and JCM) reviewed the translations to ensure that the meanings and cultural contexts were preserved. When the translations were finalized, another researcher (TL) read and coded the transcripts using MAXQDA software (15). We used a modified grounded theory approach to identify emergent themes and developed a codebook related to TB surveillance and care. TL conducted data analysis independently and met with co-investigators (RSG and JCM) midway through the analysis to discuss initial themes observed in the transcripts and reconcile disagreements. TL completed reviewing the data and developed a codebook with quotes from the transcripts to support themes. Inductive themes that emerged from the qualitative data were used to draw original and unbiased conclusions to support and interpret quantitative findings.

## Ethical considerations

A written permission letter for TB program data and to conduct the study was obtained from the NTP in Mexico City. Prior to conducting the study, the protocol was approved by the US Mexico Border Health Commission (Mexico section) Ethics Board with approval number 20-25-01-2021. Written informed consent was obtained from all focus group and in-depth interview participants. To protect the participants' privacy, study ID numbers were used instead of personal identifiers on all transcripts.

## Results

### Quantitative analysis

#### TB case notification

**Figure 2:**
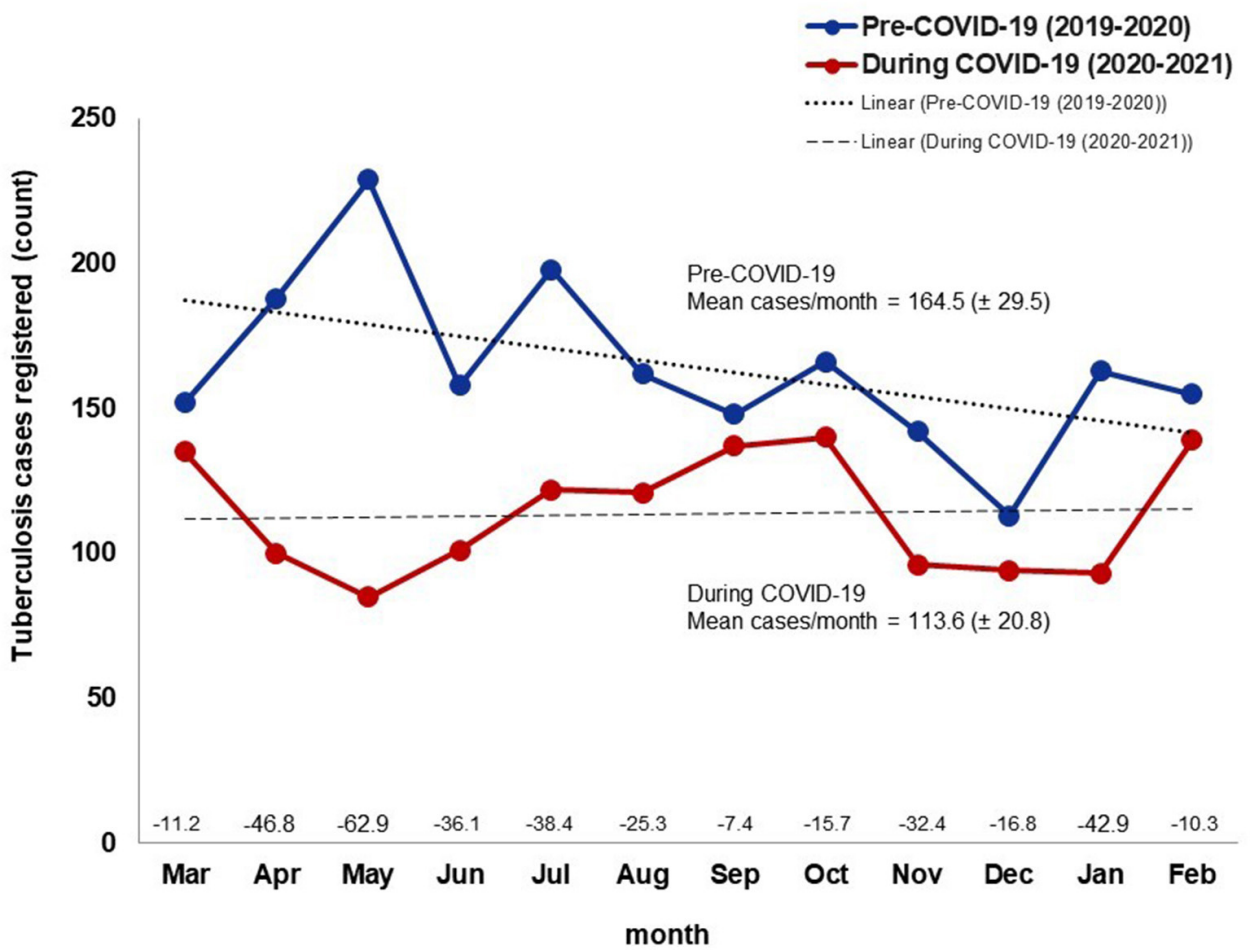
Tuberculosis cases registered by month in the year before and the first year during the COVID-19 pandemic, Baja California, México, 2019–2021. Data source: Mexico Ministry of Health management information system (https://tuberculosis.sinave.gob.mx/).

**Figure 3:**
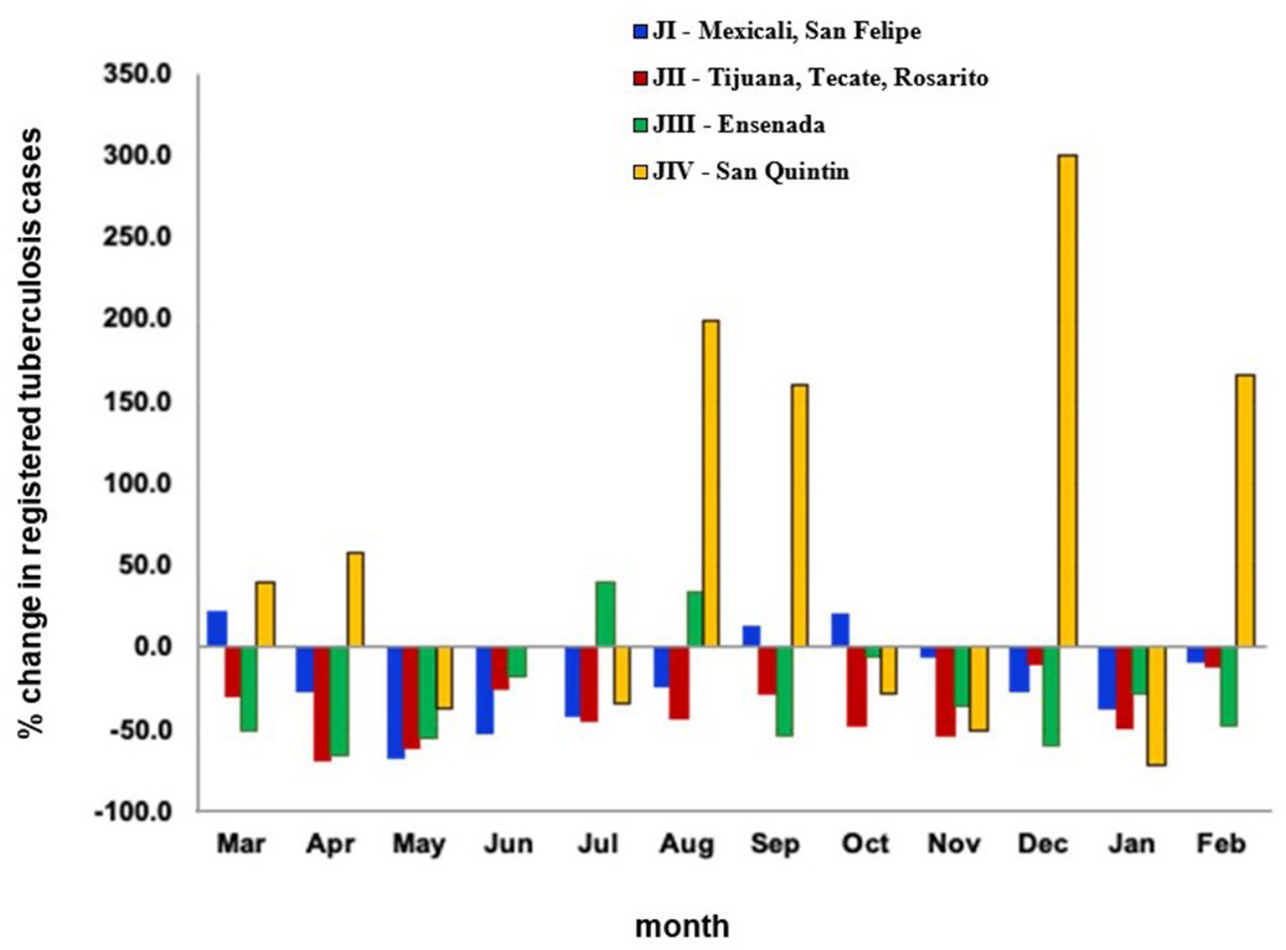
Change in the proportion of tuberculosis cases registered by month and health jurisdiction between the year before and the first year during the COVID-19 pandemic, Baja California, México, 2019–2021. Data source: Mexico Ministry of Health management information system (https://tuberculosis.sinave.gob.mx/).

The number of registered TB cases diagnosed per month declined an average of 28.9% (50.9 cases) across the four health jurisdictions in BC during the COVID-19 pandemic compared to the same time frame the year before the pandemic (Figure 2). However, in August, September, December, and February, San Quintin reported >150% increases in cases registered between pre-COVID-19 and COVID-19 periods (Figure 3).

#### TB treatment outcomes

**Figure 4:**
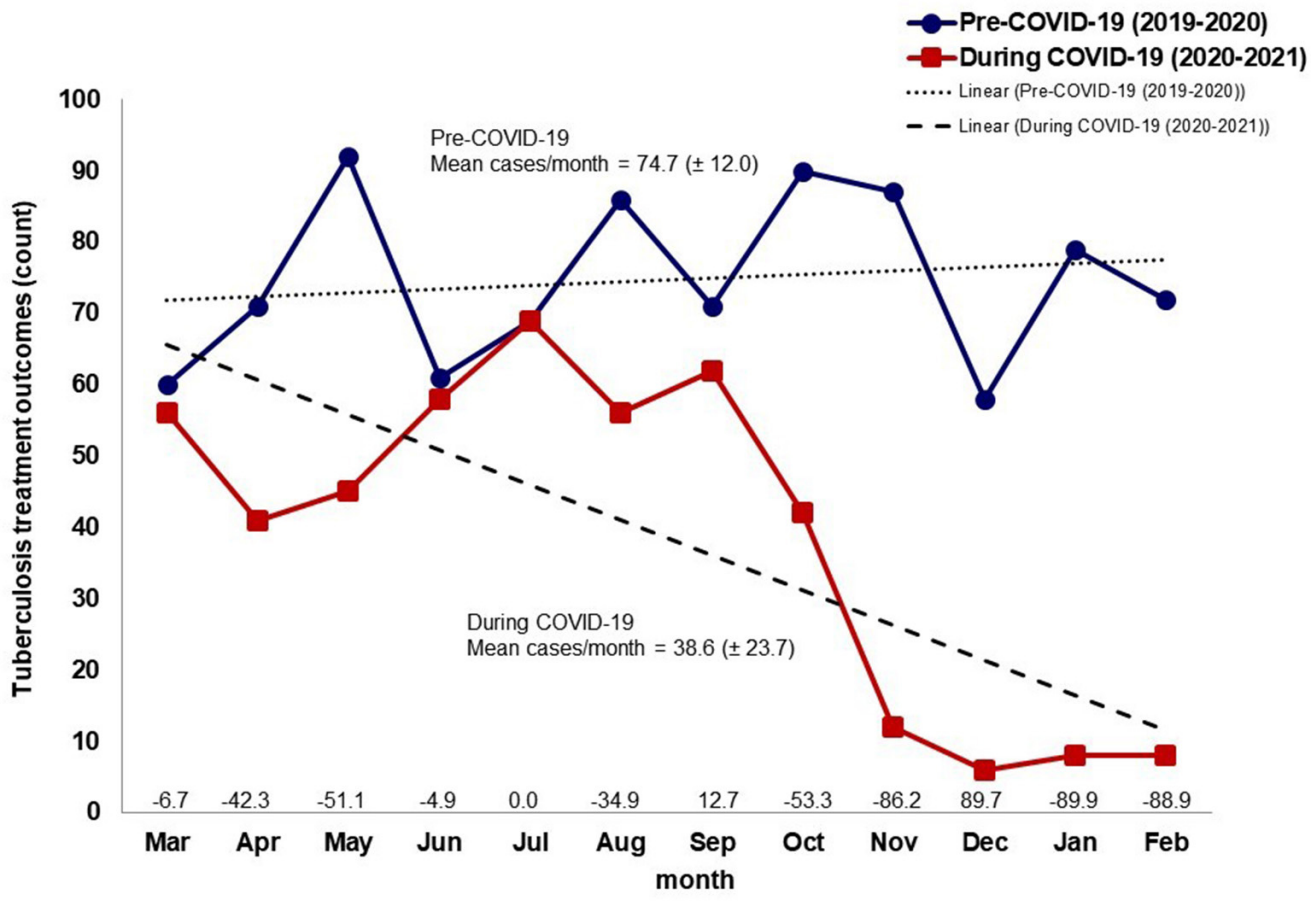
Number of tuberculosis treatment outcomes by month in the year before and the first year during the COVID-19 pandemic, Baja California, México, 2019–2021. Data source: Mexico Ministry of Health management information system (https://tuberculosis.sinave.gob.mx/).

**Figure 5:**
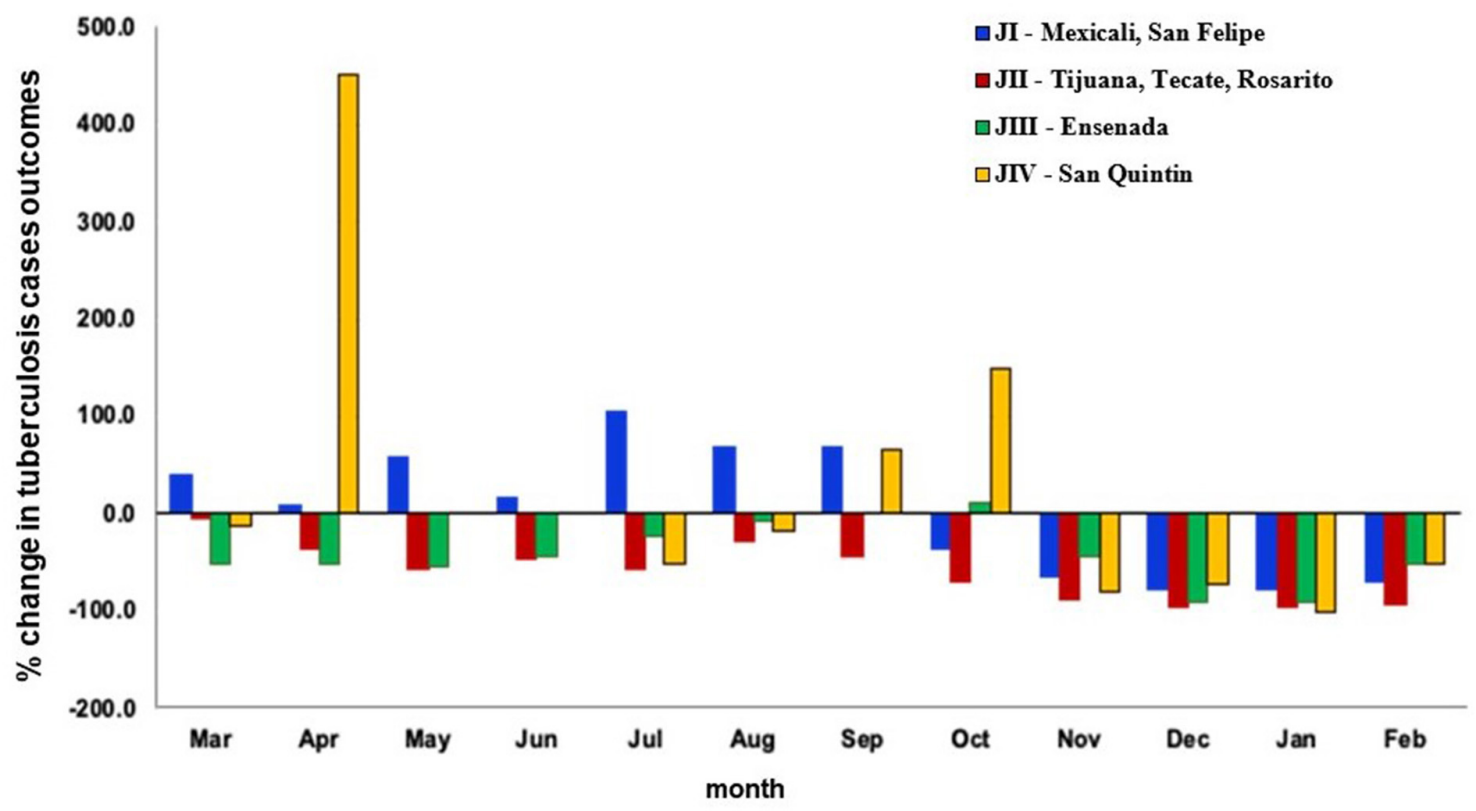
Change in the proportion of tuberculosis treatment outcomes between the year before and first year during the COVID-19 pandemic by month and health jurisdiction, Baja California, México, 2019–2021. Data source: Mexico Ministry of Health management information system (https://tuberculosis.sinave.gob.mx/).

The number of TB treatment outcomes of notified cases decreased during the COVID-19 pandemic compared to the prior year, with the greatest percentage change (−89.9%) occurring in January (Figure 4). TB treatment outcomes decreased 46.7% (38.6 cases) overall in all four health jurisdictions between the pre-COVID-19 and COVID-19 periods. Figure 5 shows that the proportion of TB treatment outcomes decreased in nearly all months for all jurisdictions during the COVID-19 pandemic compared to the year prior, except in jurisdiction IV, which showed over 100% increase in April and October.

### Qualitative analysis

**Figure 6:**
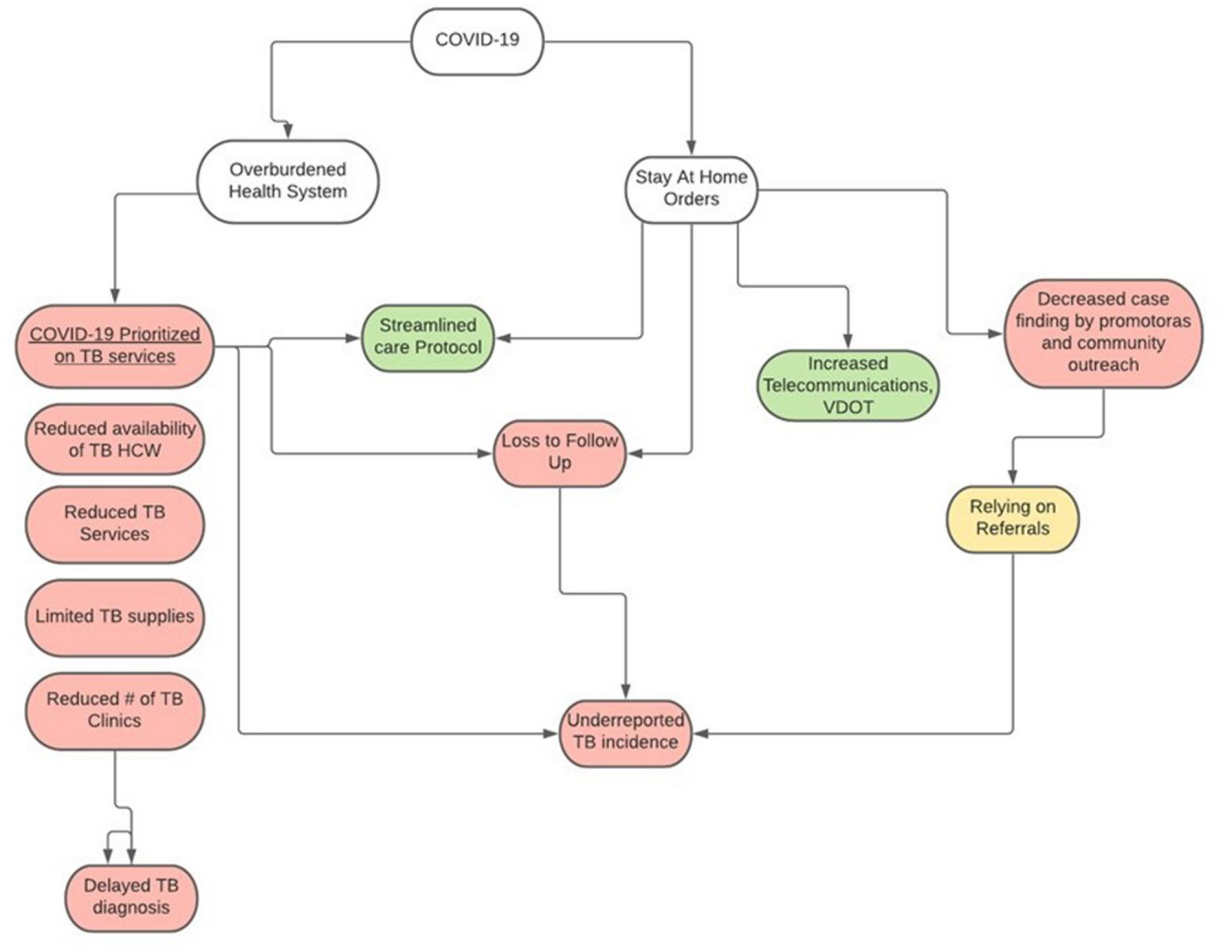
Impacts of COVID-19 pandemic on tuberculosis (TB) case detection and service delivery in Baja California, México 2021. Pink, Negative impact; Green, Positive impact.

Three in-depth interviews were conducted that included chiefs of the two main health centers in JII (Centro and Hospital General in Tijuana) and the State TB Program Coordinator. Six focus groups were conducted with a total of 43 individuals, which included 16 (37.2%) nurses, 14 (32.6%) community health workers (promotoras), 8 (18%) physicians, 4 (9.3%) Jurisdictional TB Program Coordinators, and 1 (2.3%) State TB Program Coordinator. Major themes that emerged from this analysis (Figure 6) included: decreased case finding by promotoras and community outreach, delayed TB diagnoses, reduced availability of experienced promotoras, limited access to TB supplies and services, loss to follow-up, increased telecommunications, streamlined care process, reduced number of TB clinics. TB among migrants was also discussed. These themes are described below in detail with illustrative quotes from the data.

#### Decreased case finding by promotoras and community outreach

Promotoras and nurses mentioned that community TB screening activities were curtailed during COVID-19 because safety regulations prevented them from working in highly populated areas. Instead, they limited targeted screenings to rehabilitation centers and home visits to screen household contacts of TB cases. Participants speculated that this method did not reach all TB cases, which likely resulted in an underreporting of TB prevalence.

“*We do not have promoters; the promoters are dedicated to other things, and it generates a problem of how we get the patient to come.” [JII]*

“*The truth for now, we continue with most of our colleagues in the shelter. And the priority has been on COVID vaccination. So, there is a very little outing for intentional searches in the field area. We are doing it in the city center, only with the team we have in the Jurisdiction. Because, yes, we lack in that area; to go out to the field in agricultural areas, no!” [JII]*

#### Delayed TB diagnosis

Most of the participants reported challenges with laboratory services, including a lack of laboratory diagnostic materials and a prioritization of COVID-19 testing over other tests, which caused delays in TB diagnostic services. Participants also mentioned that patients presenting respiratory symptoms had to be tested for COVID-19 before testing for TB, further delaying TB diagnostic services and treatment. The number of TB sputum smears and cultures performed decreased during COVID-19, especially between May and September 2020. However, it was noted by several participants that these problems existed prior to the COVID-19 lockdown.

“*…detection and laboratory acceptance did decrease because they also had very few personnel working. Most of the personnel were put on standby, so they always commented that they only had one laboratorian. Therefore, the number of samples received decreased.” [JI]*

“*No, no it didn't stay the same because, in the issue of respiratory problem, you know how it all was, COVID! So, it all focused on that… if the patient is and fits within the operational diagnosis of what COVID is, obviously you must go [rule out] what the pathology is and follow it up. So, in terms of [taking in patients] and all that, there have been modifications that have changed a lot; therefore, the diagnosis can sometimes take a little while to make because it's all about COVID now.” [JIII]*

#### Limited and disproportionate access to TB supplies and services

Most participants expressed their concerns about shortages on purified protein derivative tests and medications, reduced access to laboratories and X rays, and reduced access to mobile units. Lack of laboratory diagnostic materials and limited TB laboratory processing due to COVID-19 caused delays in delivery of diagnostic services. TB Program Coordinators mentioned that some health centers had to triage patients because they could not manage all the patients, which affected screening and health outcomes. Conversely, participants in a different jurisdiction reported that they did not experience problems with resources, suggesting disproportionate levels of screening, diagnosis, and treatment depending on the location.

“*The TB program at the State level is abandoned. We have been struggling to get supplies, equipment maintenance; because everything is COVID, so the money that was available for tuberculosis has been diverted to COVID, and this is going to be a problem for us.” [JII]*

“*What was not affected were the laboratory samples. The laboratory processes samples typically. Visits were not affected. The application of PPD [purified protein derivative] and PPD reading were not affected…well, the placement was not affected. Some rules were put in place to be able to continue with the admission of patients to rehabilitation centers. Some centers were not affected at all and allowed patients to enter daily; in my case, that was all.” [JI]*

#### Reduced availability of experienced TB health care workers (promotoras)

Most participants, including TB program coordinators, mentioned that the promotoras were re-assigned to support COVID-19 activities (diagnostic testing, epidemiology, and vaccination), consequently decreasing TB activities. The participants mentioned that although the TB program hired more contract workers and interns, they did not have the experience to diagnose and care for TB patients. Furthermore, since they were temporary workers, follow-up was frequently interrupted when their employment terms ended.

“*All health centers here use intern doctors due to the pandemic; the contract doctors went to work at the fever clinics, but the modules where it was not a fever clinic, as my co-worker mentioned, respiratory symptoms could not be treated. So, not just because of the pandemic! It would be good if all the modules had a permanent doctor, or at the very least a doctor contractor. So that at least, TB patients can be cared for in the place where the patient lives.” [JIV]*

“*What happened is that at one stage, I had even more contracted personnel, which was at the beginning of the pandemic, so I had a 50% reduction in personnel. Now I have fewer personnel, and of course, they ask me for fewer people, but they keep asking me for more people for the vaccination area.” (Coordinators FGD)*

#### Loss to follow up

As mentioned by most of the participants, all jurisdictions reported disruptions in directly observed therapy (DOT) for TB treatment monitoring. Stay-at-home orders, patient death, high transitory patterns, and COVID-19 screenings contributed to loss to follow up. Depending on the health system and its protocols, some systems managed to collect contact info and track down patients with TB; however, some patients in other jurisdictions were lost after being transferred to other health units.

“*When the pandemic started, we had 29 patients with MDR [multi-drug resistant TB] treatment. Of the 29, 7 died. That is almost 25%! In other words, one out of every four patients we had died during the confinement. In some of them, we knew why, and in others, we did not. We were simply notified that they had died. We did not know if it was due to COVID or not, and we lost 3, which would be about 10%. They were lost to follow-up, so the impact of the pandemic was very strong with us, as it was in the rest of the world.” [JII]*

“*And the detections were also affected, as my colleague [Male 1] said, because our colleague sometimes did not even manage to look at them because they were sent back because they had a cough; they were sent to the fever clinic. And sometimes, as [Male 1] says, they were even negative and were probably tuberculosis, but because of COVID's panic, they were sent to the fever clinic and were not diagnosed; there was no detection. That did decrease a lot too.” [JI]*

### Increased telecommunications

All participants including TB Program coordinators agreed that video directly observed therapy (i.e., patient treatment adherence monitoring delivered remotely *via* videoconference) expanded during the pandemic. The participants described that in response to prohibitions on in-person clinic or home visits, TB program staff began using synchronous video observed therapy (VOT) to monitor patient treatment adherence. Social media apps such as WhatsApp and Facebook Messenger were used to remotely observe patients swallowing their medications in real time. However, the use of VOT was limited to patients who had access to smartphones and cellular or Wi-Fi connections. Access to VOT varied by jurisdiction; San Quintin (largely rural jurisdiction) used VOT the least while Mexicali and Tijuana (largely urban) reported that 80% of TB cases used VOT.

“*So around, between jurisdiction and health centers, 80% of the cases are on video DOTS. It has worked very well for us because when we detect the patient who comes in with us, we also search for contacts with the DOTS video telephones in the jurisdiction. If the patient is taking the medication correctly, we follow up with the patient, making the patient feel a little more secure. We are calling them more constantly, responding to their videos, and they can send us a message; how they feel if they have any adverse reaction. In this way, to be able to support and orient you.” [JII]*

“*I believe that if it worked for us, I believe that now the percentage of 30-40% of people who are in video DOTS because now the study we are doing is to see the characteristics of those who comply and those who do not comply with the video DOTS.” [JIII]*

### Streamlined care processes

All participants declared that the TB care process prior to COVID-19 was complicated for patients due to administrative barriers and paperwork. During the COVID-19 pandemic, all jurisdictions in BC simplified the process by scheduling all clinic visits, X-rays, and laboratory tests to minimize the amount of time patients spent in healthcare settings. Reducing bureaucratic procedures and bringing TB services and treatment to the patient homes helped reduce financial, transportation, and access barriers for patients.

For patients receiving second-line medications for drug resistant TB, the treatment process was modified. Instead of convening a case conference with COEFAR [Comité Estatal de Farmacorresistencia], the patient's clinical information was sent to the General Hospital infectious disease specialist, who established the drug-resistant TB treatment plan and requested approval from the NTP. This modification to the administrative process reportedly reduced the time to start treatment by at least a month, and participants suggested that this change should be preserved going forward.

“*And we have also supported them directly, going to the X-ray office or to the laboratory to schedule the tests that the specialist requests in scheduling that appointment. So, I repeat, I do not know if it is an innovation, but I think it is an excellent way to give the ‘complete package' to the patient: schedule their appointment to do the tests and schedule all the examinations and laboratories they require.” [JI]*

“*We no longer required COEFAR [a reviewing committee] to analyze a patient with drug resistance… The hospital infectologist would rule or make the recommendation for treatment, and treatment was quickly requested from Mexico. We no longer paused to convening a meeting to analyze the case. This administrative process, which could take up to a month, was avoided.” [Key Informant]*

### Reduced number of TB clinics

All jurisdictions reported general challenges with routine TB screening, diagnosis, treatment, and prevention services during the COVID-19 pandemic. Reduction in clinic attendance was reported due to activity restrictions, fear of SARS-CoV-2 infection, policies restricting in-person visits, COVID-19 converted wards and entire hospitals, or complete closure of the health centers. Some patients could not attend the few health centers available because they were far from their homes, resulting in delayed diagnoses and treatment timelines, which contributed to loss to follow-up and unfavorable treatment outcomes. Some healthcare workers refused to work due to a lack of appropriate personal protective equipment, which impacted the TB services.

“*In Tijuana, almost all health centers were converted to Cl*í*nica de Fiebre [fever clinics] and/or COVID Hospital, (4 medium load centers, 2 high load centers).” [JII]*

“*The same thing the doctor was saying is practically the distance from the health centers. They are very far away from towns where only a mobile unit visits them; however, the mobile units do not carry tuberculosis treatment or does not handle TB patients. Then, the patient must be referred to a health unit, and, on some occasions, it is far away. And, if you are a patient who we can offer to bring you the treatment, we do; But now, in times of a pandemic, this option has become difficult for us.” [JII]*

#### Migrants

There were no differences between migrant and non-migrant TB services and screenings mentioned by focus group or in-depth interview participants. Participants reported using targeted screenings at rehabilitation centers to capture migrant patients; however, this method might not have captured all migrant TB patients. Tuberculosis screenings have been performed in recent migrant caravans that have come to Tijuana, but no cases of TB have been diagnosed.

Participants estimated that the number of migrant patients registered in the BC TB program is minimal, amounting to about 1–2% of the total number of cases. Participants explained that this low prevalence might be due to an increased number of transient migrants, patients concealing their migrant status, and migrant patients living in the municipality for longer than 5 years, thereby making them national cases. Participants in JI also reported difficulties with loss to follow up, particularly among US-Mexican binational patients.

“*It is difficult to estimate; when we talk about tuberculosis cases in the Jurisdiction, it never represents less than 1 or 2% of the total number of cases; however, many of them, when they approach the services, are not perceived or are not reported as migrants… The program, which we must remember, is universal. So, although we ask them for a lot of information that is collected in the epidemiological study, they may lie or carry documents that may not be completely reliable.” [JI]*

“*Since we are on the border, most of them go to work in the United States and come to live here in Mexicali. So, in our Program, 100% are binational… So, this is the only aspect in which we have had problems, that the patients have this facility, even though the checkpoint is closed; but these are patients who can cross and disappear, either from there or from here in Mexicali. So, I think this does affects us very much because we lose 100% follow-up.”[JI]*

## Discussion

Our results indicate that COVID-19 negatively impacted TB case reporting and treatment outcomes in BC, Mexico in the first year of the pandemic. We found a sharp decline in case notifications for all forms of TB during the COVID-19 outbreak compared to the year prior. Focus group discussions and in-depth interviews revealed that the main causes of the decline in TB case notification were decreased case finding by promotoras and community outreach workers, delayed TB diagnosis, and limited access to TB supplies and services during the lockdown. In contrast to these negative impacts of COVID-19, streamlined administrative processes and the increased use of telemedicine were viewed as positive outcomes from COVID-19.

All jurisdictions experienced negative impacts of the COVID-19 pandemic, ranging from minor to very significant during at least 1 month during COVID-19. TB clinics seemed to be most severely affected in May 2020, 2 months into the lockdown. Participants from all jurisdictions reported problems with obtaining adequate supplies and reagents, as well as with adapting to new requirements like physical distancing or working remotely. Staff shortages linked to lockdowns, isolation and quarantine, and relocation to COVID-19 units affected TB operations, especially in March and April of 2020. All jurisdictions experienced difficulties from personnel being re-allocated to COVID-19 vaccination services. TB diagnostic services and TB outcomes were severely affected, with the disruption peaking in May 2020 reported by all jurisdictions. The pandemic response also led to longer TB testing turnaround times, as well as suspension of diagnostics and treatment services. The number of TB notifications gradually improved after December 2020 as services were adapting to a “new normality phase.”

Incidence of TB in 2020 was lower than 2019 nationally in Mexico and in BC (8, 9). While this may have been due to COVID-19 mitigation protocols, reduced laboratory testing and potential misclassification from TB patients reported as COVID death may have also driven this decline. A key informant revealed that they were unable to determine the cause of death for all their patients, suggesting that there may have also been cases of TB that died without being diagnosed.

Qualitative analysis revealed that patients presenting respiratory symptoms were redirected to “fever clinics” for SARS-CoV-2 screening. Patients who were negative for COVID-19 did not receive further testing and were often lost to follow-up, especially if they were transferred to fever units in other hospitals. Overburdened health systems from COVID-19 and lack of coordination among health systems contributed to loss to follow up and potentially misclassified cause of death.

Participants from San Quintin (JIV) and Ensenada (JIII) reported being able to follow all cases with and without COVID-19 symptoms; consequently, San Quintin reported higher TB notifications during COVID-19 in comparison to pre-COVID-19. Compared to the other jurisdictions, San Quintin is geographically dispersed and known for its agricultural export market (16). About 80% of the workforce in San Quintin is migrant, of which 54.5% are permanent migrants who have worked there for several years and 45.5% are temporary (17). Participants from San Quintin mentioned that they screened migrants for TB when they arrived to work on the farms, but this practice was suspended during the pandemic. Qualitative analysis did give insight on why registered TB in San Quintin increased during the pandemic; TB program personnel from this jurisdiction conducted TB screening in addition to the COVID-19 survey for patients with cough with phlegm. In addition, they performed diagnostic TB tests.

Mexico is not unique in terms of the effects of COVID-19 on TB. According to a global survey, 25 of 44 countries had introduced changes to TB service delivery since the start of the COVID-19 pandemic, with 10 countries and nine countries reducing the number of in-patient and outpatient TB facilities, respectively (7). Like BC, challenges were also reported by TB professionals in both West African and European settings, especially regarding staff shortages and laboratory issues that predated COVID-19 (18). Consequently, TB incidence and TB mortality are projected to increase by 5–15% over the next 5 years, resulting in hundreds of thousands of additional TB deaths worldwide (19, 20). COVID-19 adaptations found in our study were similar to mitigation strategies used in other counties, such as reduced frequency of outpatient visits for treatment monitoring or drug dispensing, allowing TB patients to take a 1-month or more supply of anti-TB drugs home, expanded use of remote advice and support, and home delivery of anti-TB drugs (7).

Although immigration records suggest that more than 50% of the population in BC is migrant (21), there are sparse data about the migration status of TB cases in any jurisdiction in BC (8, 9). However, according to the participants of this study, 1 or 2% of the total number of TB cases in BC are migrant. Similarly, in California, 2% of TB patients were reported as migrant or seasonal worker pre-COVID-19 and during COVID- (22). Migration status information is important for TB programs to assess because migrants are at higher risk of communicable diseases, traumatic events, inadequate health care, etc. (23). Likewise, limited political visibility and protection for migrants further deteriorates their health, as seen during the pandemic when they disproportionately shouldered COVID-19 morbidity and mortality in 2020 (24). Identifying migrant patients and understanding their health is key to reducing mortality and improving population health.

## Strengths and limitations

Migrant status data were not available for any jurisdiction, so we were unable to determine differences in TB treatment outcomes for migrant patients. TB burden among migrant populations in BC remains unclear, especially during the pandemic. Furthermore, this study does not reflect the perspectives of patients with TB during this time, which may have given insight on the quality and reach of these TB services. In addition, only healthcare workers were included in the qualitative phase of this study; thus, our findings are limited to provider perspectives on how COVID-19 impacted TB diagnosis and treatment. Despite these limitations, we believe our study provides first-hand experiences of TB healthcare workers and program coordinators in BC during the pandemic.

## Conclusion and future directions

This is the first study conducted in BC, Mexico to describe the impact of COVID-19 on the State TB Program performance. This study provides novel information about TB program strategies implemented in response to the COVID-19 pandemic that may have affected migrants in BC, Mexico. It will also identify additional TB program functions that should be addressed to better serve all TB patients, including migrants well beyond the COVID-19 pandemic. These findings will assist TB program directors at the local, state, and federal levels in Mexico to make evidence-based decisions around TB program policies.

TB control professionals in BC experienced challenges in delivering TB diagnosis and treatment services due to the COVID-19 pandemic and this study highlights the need for clear communication of guidelines, prioritization of routine TB service delivery, ongoing health education, and possible integration of TB and COVID-19 services to ensure that TB services are more resilient against the impact of this respiratory disease pandemic. Migrants with TB are potentially disproportionately impacted by COVID-19 due to crowded living conditions, reduced health screenings, and increased risk of loss to follow-up; however, migration status is not uniformly assessed by TB programs, making it difficult to fully understand their situation. Some positive TB program changes were noted, such as an increase in the use of telemedicine and a streamlined process for initiating treatment for patients with drug resistant TB, which programs should consider maintaining after the pandemic ends.

## Data availability statement

The raw data supporting the conclusions of this article will be made available by the authors, without undue reservation.

## Author contributions

RM-S and RSG conceived of, designed, and drafted the manuscript. RM-S, RSG, TL, JC-M, and JG-F conducted the qualitative analysis. RM-S, JG-F, RZ-G, JR-F, and PR-T contributed to quantitative data analysis. All authors contributed to the interpretation of data, revision of the manuscript for important intellectual content, and have read and approved of the final version of the manuscript.

## Funding

This work was funded by Research Program on Migration and Health (PIMSA, 2020) awarded to RM-S and RSG.

## Conflict of interest

The authors declare that the research was conducted in the absence of any commercial or financial relationships that could be construed as a potential conflict of interest.

## Publisher's note

All claims expressed in this article are solely those of the authors and do not necessarily represent those of their affiliated organizations, or those of the publisher, the editors and the reviewers. Any product that may be evaluated in this article, or claim that may be made by its manufacturer, is not guaranteed or endorsed by the publisher.
